# RETRACTION: PEGylated Polyethylenimine Derivative-Mediated Local Delivery of the shSmad3 Inhibits Intimal Thickening after Vascular Injury

**DOI:** 10.1155/bmri/9808963

**Published:** 2025-07-30

**Authors:** BioMed Research International

RETRACTION: Y. Wang, D. Zhao, X. Wei, L. Ma, J. Sheng, and P. Lu, “PEGylated Polyethylenimine Derivative-Mediated Local Delivery of the shSmad3 Inhibits Intimal Thickening after Vascular Injury,” *BioMed Research International* 2019 (2019): 8483765, https://doi.org/10.1155/2019/8483765.

The above article, published online on 29 July 2019 in Wiley Online Library (http://wileyonlinelibrary.com), has been retracted following the agreement the Section Editor of the journal Letterio Politi; and John Wiley & Sons Ltd.

The retraction has been agreed following an investigation of the concerns raised by *Daviesia triflora* and *Bulbothrix tabacina* on PubPeer [1].

More specifically, the investigation has identified unexpected similarities in the subpanels *b*, *c*, and *d* of Figure 8a, which represent different experimental conditions.

As a result of the investigation, the data and conclusions of this article are considered unreliable.

The authors disagree with this retraction.
